# Deep Venous Thrombosis and Risk of Consequent Sepsis Event: A Retrospective Nationwide Population-Based Cohort Study

**DOI:** 10.3390/ijerph18157879

**Published:** 2021-07-25

**Authors:** Ying-Tung Yeh, Sheng-En Tsai, Ying-Cheng Chen, Shun-Fa Yang, Han-Wei Yeh, Bo-Yuan Wang, Liang-Tsai Yeh, Nai-Chen Shih, Yu-Hsun Wang, Yin-Yang Chen, Chao-Bin Yeh

**Affiliations:** 1 Graduate School of Dentistry, School of Dentistry, Chung Shan Medical University, Taichung 40201, Taiwan; cshy236@csh.org.tw; 2 Department of Dentistry, Chung Shan Medical University Hospital, Taichung 40201, Taiwan; 3 Department of Anesthesiology, Changhua Christian Hospital, Changhua 50006, Taiwan; 176500@cch.org.tw (S.-E.T.); 68990@cch.org.tw (L.-T.Y.); 4 Institute of Medicine, Chung Shan Medical University, Taichung 40201, Taiwan; 137448@cch.org.tw (Y.-C.C.); ysf@csmu.edu.tw (S.-F.Y.); cshy1473@csh.org.tw (B.-Y.W.); 5 Department of Surgery, Changhua Christian Hospital, Changhua 50006, Taiwan; 6 Department of Medical Research, Chung Shan Medical University Hospital, Taichung 40201, Taiwan; cshe731@csh.org.tw; 7 School of Medicine, Chang Gung University, Taoyuan City 33302, Taiwan; b0502016@stmail.cgu.edu.tw; 8 Chang Gung Memorial Hospital, Linkou, Taoyuan City 33302, Taiwan; 9 Department of Emergency Medicine, School of Medicine, Chung Shan Medical University, Taichung 40201, Taiwan; 10 Department of Emergency Medicine, Chung Shan Medical University Hospital, Taichung 40201, Taiwan; 11 Department of Family Medicine, Taichung Veterans General Hospital, Taichung 40705, Taiwan; u100001303@cmu.edu.tw; 12 Department of Surgery, Chung Shan Medical University Hospital, Taichung 40201, Taiwan

**Keywords:** sepsis, deep vein thrombosis, cohort study

## Abstract

Deep vein thrombosis causes several acute and chronic vessel complications and puts patients at risk of subsequent sepsis development. This unique study aimed to estimate the risk of sepsis development in DVT patients compared with non-DVT patients. This population-based cohort study used records of a longitudinal health insurance database containing two million patients defined in Taiwan’s National Health Insurance Research Database (NHIRD). Our study included patients aged over 20 years with a new diagnosis of DVT with at least two outpatient department visits or an admission between 2001 and 2014. Patients with a diagnosis of sepsis before the index date were excluded. Propensity score matching (PSM) was used to homogenize the baseline characteristics between the two groups. To define the independent risk of the DVT group, a multivariate Cox proportional hazard model was used to estimate the hazard ratios. After PSM, the DVT group (*n* = 5753) exhibited a higher risk of sepsis (adjusted hazard ratio, aHR, 1.74; 95% CI, 1.59–1.90) compared with non-DVT group (*n* = 5753). Patients with an increased risk of sepsis were associated with being elderly aged, male, having diabetes, chronic kidney disease, chronic obstructive pulmonary disease, stroke, malignancy, and use of antibiotics. In conclusion, this population-based cohort study demonstrated an increased risk of sepsis in DVT patients compared with non-DVT patients. Thus, early prevention and adequate treatment of DVT is necessary in clinical practice.

## 1. Introduction

Deep vein thrombosis (DVT) is a clinical manifestation of venous thromboembolism (VTE) and can contribute to severe morbidity, mortality, and economic burden as well. Even though often underestimated, there were more than 200,000 deep vein thrombosis cases annually in the United States [1] and about 500,000 in European countries [2]. In Taiwan, the crude incidence of VTE was 15.9 per 100,000 person-years, with a higher risk of recurrence in people with a history of VTE, cancer, major extremity trauma, or undergoing major surgery [3]. The economic burden of the initial VTE is approximately $3000–9500, equivalent to $33,000 for each individual [4] and estimated to be $7–10 billion in the United States [5].

The major theory illustrating the pathogenesis of DVT, known as the Virchow’s triad, includes three main mechanisms of venous stasis, endothelial damage, and a hypercoagulation state [6]. Clinical presentations of DVT are nonspecific, and many patients are asymptomatic. However, DVT should be suspected in patients who present with classical symptoms of pain, warmth, leg swelling, and erythema. The common risk factors of DVT include older age, history of DVT, obesity, active cancer [7], hormone replacement therapy [8], surgery [9], trauma, hospitalization, nursing home residence, and paralysis [10]. Recently, DVT has also been considered a complication after vaccination for coronavirus disease 2019 (COVID-19) [11].

On the other hand, when dealing with patients with sepsis, especially those complicated by hypotension and septic shock in clinical practice, DVT was often considered one of those troublesome complications [12,13]. Kaplan et al. performed a multicenter prospective study and showed a 37.2 (95% CI, 28.3–46.8) incidence of VTE in patients with severe sepsis and septic shock, despite using guideline-recommended medications for thromboprophylaxis [14]. Aside from the acting complications of sepsis, infection and sepsis resulting from DVT is an important issue but infrequently mentioned and studied.

DVT causes both acute and chronic complications associated with sepsis development. The consequence of chronic venous insufficiency resulting from DVT, manifesting as mild limb swelling, intractable edema, to severe leg venous ulceration, is known as post-thrombotic syndrome (PTS) [15]. Even under adequate anticoagulant medications, PTS develops in about 20% to 50% of DVT patients [16]. Chronic venous ulceration could further develop a local infection, cellulitis, and even sepsis. In contrast to the chronic complication of DVT, acute DVT could also cause an emergent condition called phlegmasia cerulea dolens (PCD) [17]. PCD is a life-threatening complication of acute DVT, with cascading symptoms from extremities swelling and pain, pale, tissue ischemia, cyanosis to necrosis and sepsis, especially when accompanied with compartment syndrome [18].

Nevertheless, most studies focused on the risk stratifications of DVT and prevention strategies rather than assessment risk of subsequent infection or even sepsis caused by DVT. Presently, no studies have conducted an evaluation of the risk of sepsis in patients with DVT or subgroups linked with a higher risk and poor prognosis due to sepsis. Hence, this study examined whether DVT increases the risk of sepsis and their complications by executing a present nationwide population-based cohort study using the National Health Insurance Research Database (NHIRD).

## 2. Materials and Methods

### 2.1. Data Sources

The longitudinal health insurance database is regulated by the Health and Welfare Data Science Center (HWDC) in Taiwan. The database contains two million beneficiaries that were randomly sampled from the 2000 registry for beneficiaries out of the entire population. The database contains all outpatients’ and inpatients’ medical claims, as well as details on drugs prescribed, medical operations, procedures, and medical expenses from 2000 to 2015. The cause of death data contains the code and date of death and has been validated by previous research [19]. The study was approved by the ethical review board of the Chung Shan Medical University Hospital (CS1-20056).

### 2.2. Study Design and Outcome

This study is a retrospective cohort study design with a study population based on new diagnoses of deep vein thrombosis (DVT; ICD-9-CM codes = 453.8) and age ≥ 20 years from 2001 to 2014. In order to ensure the accuracy of the diagnoses, outpatient visits of ≥ 2 times or hospitalizations of ≥ 1 time were imposed. The index date was established as the first diagnosis date of deep vein thrombosis. The positive predictive value of DVT codes used in this study was about 90%, as shown in a previous validation study [20]. Diagnoses of sepsis (ICD-9-CM = 038, 995.91, 995.92) before the index date were excluded to ensure new DVT onset patient samples. The non-DVT subjects were defined with no diagnosis of deep vein thrombosis from 2000 to 2015. The outcome variable was defined as a diagnosis of sepsis (ICD-9-CM = 038, 995.91, 995.92) from emergency or admission. A previous validation study also showed the codes used to define sepsis in this study obtained a positive predictive value of 75% (95% CI, 53.0–90.2%) and a negative predictive value of 99.4% (95% CI, 98.3–99.8%) [21]. Both groups were followed up until the onset of sepsis, death, or 31 December 2015, whichever occurred first.

### 2.3. Covariates and Matching

The baseline characteristics were age, sex, hypertension (ICD-9-CM = 401–405), hyperlipidemia (ICD-9-CM = 272.0–272.4), diabetes (ICD-9-CM = 250), ischemic heart disease (ICD-9-CM = 410–414), chronic kidney disease (ICD-9-CM = 585), chronic obstructive pulmonary disease (ICD-9-CM = 491, 492, 496), intracranial bleeding (ICD-9-CM = 430–432), stroke (ICD-9-CM = 433–438), malignancy (ICD-9-CM = 140–208), rheumatoid arthritis (ICD-9-CM = 714.0), *systemic lupus erythematosus* (SLE, ICD-9-CM = 710.0), Sjogren’s syndrome (SLE, ICD-9-CM = 710.2), ankylosing spondylitis, (ICD-9-CM = 720.0), psoriasis (ICD-9-CM = 696.0, 696.1), and peripheral arterial occlusive disease (PAOD, ICD-9-CM = 443.8, 443.9, 444). These comorbidities were defined before the index date within one year and at least two outpatient visits or one hospitalization. In addition, antibiotics use before the index date, within one year and ≥ 4 times orders were included. The accuracy and positive predictive value of the diagnosis of covariate diseases in the NHIRD has previously been validated [22].

**Figure 1:**
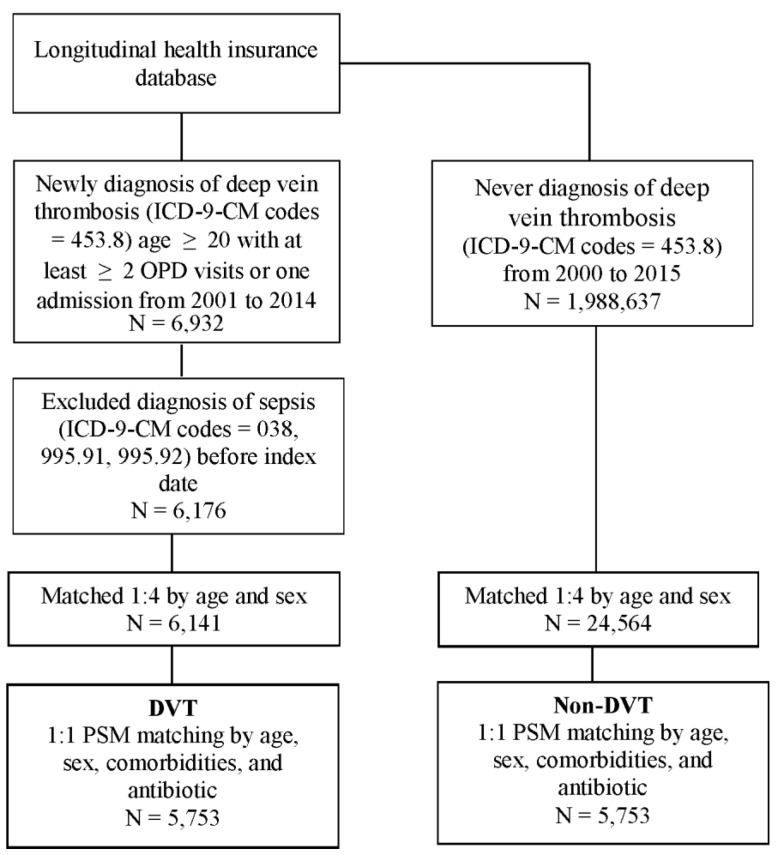
Flowchart of patient selection.

First, a 1:4 matching by age and sex was used to provide an index date for the non-DVT subjects that had the same starting point. Then, propensity score matching was performed by age, sex, comorbidities, and antibiotics use between the two groups (Figure 1). The propensity score was a probability that was estimated through logistic regression. The binary variable was the DVT and non-DVT groups. By matching the propensity score, it could balance the heterogeneity of the two groups.

### 2.4. Statistical Analysis

Comparison of the DVT group and non-DVT group was performed using absolute standardized differences (ASD). When the absolute standardized difference was less than 0.1, it was defined as the characteristics of both groups being similar [23]. The relative risk (RR) and 95% confidence intervals (CI) were calculated via the Poisson regression model. Kaplan–Meier analysis was used to calculate the cumulative incidence of sepsis among the two groups. The log-rank test was used to test the significance. To identify the independent risk of the DVT group, a multivariate Cox proportional hazard model was used to estimate the hazard ratios. We performed E-value to define the minimum strength of the association for an unmeasured confounding effect between deep venous thrombosis and sepsis [24]. The statistical software was SAS version 9.4 (SAS Institute Inc., Elizabeth City, NC, USA). 

## 3. Results

### 3.1. Characteristics of the Study Subjects

We identified 6932 patients who were newly diagnosed with DVT via outpatient visit or admission from 2001 to 2014. After applying exclusion criteria and 1:4 age and sex matching, there were 6141 DVT patients versus 24,564 non-DVT patients. The DVT groups showed a higher proportion of elderly and female patients. Compared with the non-DVT group, the DVT group had a higher proportion of comorbidities, including hypertension, hyperlipidemia, diabetes, ischemic heart disease, chronic kidney disease, stroke, malignancy, and peripheral arterial occlusive disease. The DVT group also had a higher proportion of antibiotics use. We further performed PSM and conclusively included 5753 DVT patients with 5753 control cases for examination. A comparison of the characteristics of the DVT and non-DVT patients is presented in Table 1. Age was on the borderline with no significant difference between the PSM-derived DVT and non-DVT groups (ASD = 0.10). Hypertension was the comorbidity with the highest prevalence in both groups, followed by diabetes, ischemic heart disease, and hyperlipidemia. Sex, comorbidities, and antibiotics use were not significantly different between the PSM-derived DVT and non-DVT groups.

### 3.2. Risk of Sepsis between DVT and Non-DVT Group

**Figure 2:**
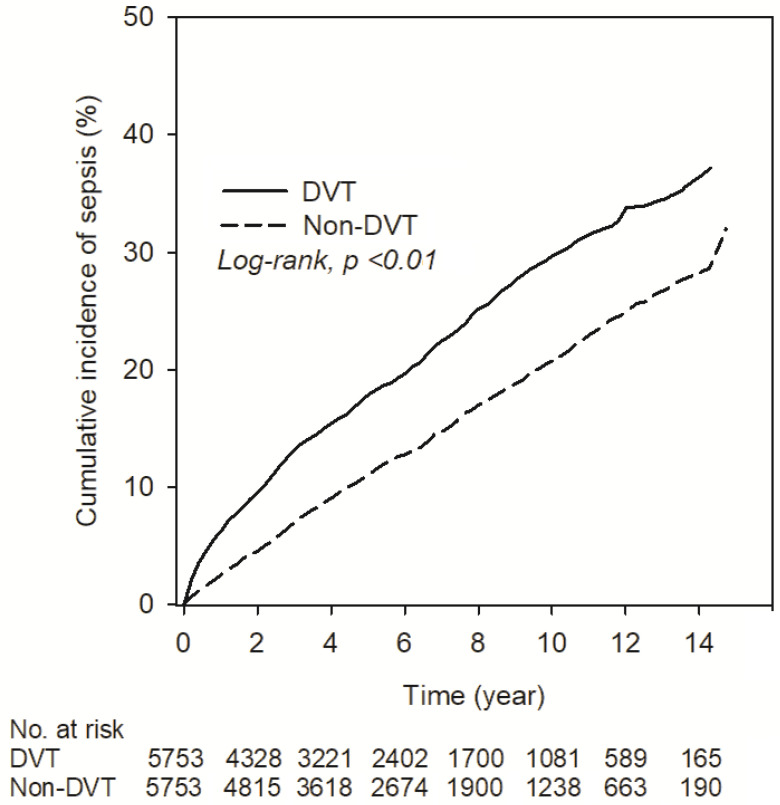
Kaplan–Meier curves of the cumulative proportions of sepsis in DVT patients.

After PSM, the incidence of sepsis was 37.25 (95% CI, 35.20–39.41) and 23.59 (95% CI, 22.06–25.24) in the DVT and non-DVT cohorts, respectively. The cohort with DVT presented an increased incidence of sepsis (RR, 1.58; 95% CI, 1.45–1.72) (Table 2). The Kaplan–Meier survival analysis showed a significantly higher cumulative incidence of sepsis in the DVT group (Log-rank, *p* <0.01) (Figure 2). Compared with the patients in the non-DVT group, those with DVT presented an increased risk of sepsis (aHR, 1.74; 95% CI, 1.59–1.90). Other significant risk factors of sepsis included older age, male, diabetes, chronic kidney disease, COPD, stroke, malignancy, and use of antibiotics (Table 3). In contrast, hyperlipidemia showed a protective effect on sepsis. As shown in Table 4, subgroup analysis performed on the association of DVT with a significantly higher risk of sepsis included the stratification of age, sex, hypertension, hyperlipidemia, diabetes, ischemic heart disease, chronic kidney disease, COPD, stroke/TIA, malignancy, and antibiotics use. For patients aged 20–39 years in the non-hypertension and non-antibiotic subgroup, DVT subjects performed a significantly higher risk of sepsis than other subgroups (*p* for interaction <0.01, <0.01, 0.03, respectively). The E-value of the analyses was 2.29 and performed the minimum strength of an unmeasured confounder.

## 4. Discussion

This is the first nationwide population-based cohort study conducted to clarify the risk of sepsis in DVT patients, and the findings revealed that DVT is associated with a higher risk of sepsis (aHR, 1.74; 95%CI; 1.59–1.90). In the past decades, DVT was considered a troublesome complication of sepsis and septic shock [11,12]. We discovered an inverse relationship between DVT and sepsis and provided an alternative thinking process for clinical practice. Since most previous studies have emphasized prevention and early medication of DVT with other diseases [25], our study showed the importance of the prevention of infection and sepsis in DVT patients.

Our findings are compatible with the pathophysiology of DVT to PTS, extending to sepsis caused by venous leg ulcers [26,27]. It is estimated that more than 20% of DVT patients develop a chronic clinical presentation of local or segmental edema, cramping pain, redness, hyperpigmentation of the painful area, and eventually ulceration [28]. Two mechanisms are responsible for venous hypertension and the progression from DVT to PTS, including venous obstruction and valvular incompetence due to vein valves damage [15]. PTS is the long-term complication of DVT and causes chronic socioeconomic morbidity [29].

For clinical diagnosis and severity evaluation of PTS, the Villalta scale has been adopted by the International Society on Thrombosis and Haemostasis [30] and is applied worldwide [31] due to its validity and reproducibility throughout the disease process. The scale contains five symptoms and six clinical signs, with each component scored from 0 to 3. Recently, several potential novel biomarkers have been proposed for improving the diagnosis of DVT and PTS [32], including intracellular adhesion molecule-1(ICAM-1) [33], P-selectin [34], and cell-free DNA [35].

Malignancy is a risk factor of both DVT and sepsis, as described and proved in a previous study [36,37]. Our study showed a similar result that malignancy was associated with a higher risk of sepsis (aHR, 1.75; 95%CI, 1.55–1.99). Moreover, our study performed subgroup analysis and found that for both non-malignancy and malignancy groups, patients with DVT in both subgroups obtained a higher risk of sepsis (aHR, 1.74, 2.15, respectively). Compared with non-malignancy patients, malignancy patients with DVT were associated with a slightly greater but not significantly higher risk of sepsis (*p* for interaction = 0.11).

On the other hand, peripheral arterial occlusive disease (PAOD) is another common vascular disease of distal extremities, causing local pain, ischemia, necrosis, wound infection, spies, and potentially results in amputation. Studies have shown a positive correlation between PAOD and infection [38]. However, PAOD in our study showed a borderline increased risk of sepsis in the univariate model (aHR, 1.41; 95%CI, 1.13–1.76) but a decreased risk of sepsis in the multivariate model (aHR, 0.97; 95%CI, 0.77–1.21). One possible explanation could be the improvement of diagnostic tools, medication, and endovascular intervention of PADO, resulting in less severity of disease progression.

Our study also demonstrated that younger DVT patients were susceptible to a higher risk of sepsis (*p* for interaction = 0.0093). While the incidence of DVT in young adults is rare [39], the development of DVT at a younger age is related to thrombophilic disorders and anomalies of the inferior vena cava (IVC) [40]. IVC anomalies could affect the blood return of the lower extremities and increase the condition of venous stasis and venous hypertension [41], while thrombophilia is a hypercoagulation state due to the over-activity of coagulation factors. However, the higher correlation between younger DVT patients and sepsis compared with older DVT patients may be due to the different distribution of co-infection between the age subgroups, and more studies are required for a plausible mechanism and confirmation. In addition, this study result showed the protective effect of hyperlipidemia against sepsis, which could be explained as the statins prescriptions for hyperlipidemia patients. Previous studies have demonstrated a similar correlation that statins use was associated with a lower risk of developing sepsis [42,43,44].

Based on the results of our study, the early management of DVT and prevention for sepsis are essential in clinical practice. Multidisciplinary treatment and consultation are necessary, including anticoagulant therapy of low-molecular-weight heparin and non-vitamin K antagonist oral anticoagulant (NOAC), elastic compression stockings [45], infection controls, adequate wound dressing, and a surgical or endovascular approach [46].

This observational study had some limitations. First, the NHIRD did not obtain information on the severity of DVT, patients’ activity ability or patients’ personal behaviors, such as smoking and obesity, or factors that influence wound healing and risk of thrombosis. Second, the database also lacked the details of initial treatment of DVT and therapy procedures, such as medication treatment or indicated surgical intervention. Third, co-existed infection was not excluded in the study population; however, we used PSM for antibiotics use in the DVT and non-DVT group analysis to minimize potential bias. Fourth, PTS was a set of signs and symptoms and was not defined in the ICD-9-CM codes system, resulting in a lack of PTS data in our study. However, DVT patients developed sepsis mainly via PTS. Since the correlation mechanism between DVT, PTS, and sepsis were defined, the lack of PTS data in our results did not affect the main outcome of the study. Finally, this observational study extracted data from Taiwan’s national database. More precisely designed and planned studies with randomized control trials are warranted for further analysis and evaluation of the risk of DVT from sepsis before worldwide application.

## 5. Conclusions

In summary, this population-based cohort study with PSM demonstrated that DVT is associated with an increased risk of sepsis, with a protective effect against sepsis in patients of older age, with hypertension, and under antibiotics use. Early intervention and prevention of sepsis complications are essential for clinical practice when caring for DVT patients.

## Figures and Tables

**Table 1:** Demographic characteristics of DVT and non-DVT patients.

	After PSM Matching									
Variables	Non-DVT (*N* = 24,564)		DVT (*N* = 6141)			Non-DVT (*N* = 5753)		DVT (*N* = 5753)		
	*n*	%	n	%	ASD	n	%	n	%	ASD
Age					0					0.10
20–39	2216	9.0	554	9.0		463	8.0	521	9.1	
40–64	10,164	41.4	2541	41.4		2146	37.3	2330	40.5	
≥65	12,184	49.6	3046	49.6		3144	54.6	2902	50.4	
Mean ± SD	63.19 ± 15.87	63.19 ± 15.87			0	64.88 ± 15.71	63.35 ± 15.95	0.10
Sex					0					0.01
Female	14,244	58.0	3561	58.0		3359	58.4	3327	57.8	
Male	10,320	42.0	2580	42.0		2394	41.6	2426	42.2	
Hypertension	7846	31.9	2730	44.5	0.26	2621	45.6	2470	42.9	0.05
Hyperlipidemia	2942	12.0	951	15.5	0.10	940	16.3	877	15.2	0.03
Diabetes	3372	13.7	1246	20.3	0.18	1171	20.4	1116	19.4	0.02
Ischemic heart disease	2368	9.6	1030	16.8	0.21	975	16.9	905	15.7	0.03
Chronic kidney disease	379	1.5	660	10.7	0.39	376	6.5	438	7.6	0.04
COPD	1349	5.5	461	7.5	0.08	489	8.5	433	7.5	0.04
Intracranial bleeding	136	0.6	85	1.4	0.09	74	1.3	71	1.2	0.01
Stroke/TIA	1686	6.9	693	11.3	0.15	682	11.9	633	11.0	0.02
Malignancy	923	3.8	854	13.9	0.36	746	13.0	695	12.1	0.03
Rheumatoid Arthritis	129	0.5	72	1.2	0.07	62	1.1	61	1.1	<0.01
SLE	18	0.1	42	0.7	0.10	17	0.3	14	0.2	0.01
Sjogren’s syndrome	112	0.5	49	0.8	0.04	35	0.6	40	0.7	0.01
AS	24	0.1	21	0.3	0.05	14	0.2	14	0.2	0.01
Psoriasis	61	0.2	27	0.4	0.03	22	0.4	25	0.4	<0.01
PAOD	222	0.9	249	4.1	0.20	164	2.9	183	3.2	0.02
Antibiotics use	3516	14.3	1886	30.7	0.40	1697	29.5	1630	28.3	0.03

DVT, deep vein thrombosis; ASD, absolute standardized differences; COPD, chronic obstruction pulmonary disease; TIA, transient ischemic attack; SLE, systemic lupus erythematosus; AS, ankylosing spondylitis; PAOD, peripheral arterial occlusion disease.

**Table 2:** Poisson regression of relative risk of DVT and non-DVT patients.

Variables	Non-DVT	DVT
N	5753	5753
Person-years	35,860	32,273
No. of sepsis	846	1202
ID (95% CI)	23.59 (22.06–25.24)	37.25 (35.20–39.41)
Relative risk (95% CI)	Reference	1.58 (1.45–1.72)

ID, incidence density (per 1000 person-years); DVT, deep vein thrombosis; CI, confidence interval.

**Table 3:** Cox proportional hazard model analysis for risk of sepsis.

	Univariate		Multivariate †	
Variables	HR (95% CI)	*p*-Value	HR (95% CI)	*p*-Value
Group				
Non-DVT	Reference		Reference	
DVT	1.57 (1.44–1.71)	<0.01	1.74 (1.59–1.90)	<0.01
Age				
20–39	Reference		Reference	
40–64	2.25 (1.67–3.04)	<0.01	1.96 (1.45–2.65)	<0.01
≥65	7.96 (5.96–10.65)	<0.01	5.69 (4.22–7.67)	<0.01
Sex				
Female	Reference		Reference	
Male	1.31 (1.20–1.43)	<0.01	1.12 (1.03–1.23)	0.01
Hypertension	2.01 (1.84–2.20)	<0.01	1.05 (0.96–1.16)	0.29
Hyperlipidemia	1.14 (1.01–1.28)	0.04	0.83 (0.73–0.94)	<0.01
Diabetes	2.23 (2.02–2.45)	<0.01	1.59 (1.44–1.76)	<.001
Ischemic heart disease	1.77 (1.60–1.96)	<0.01	1.10 (0.99–1.22)	0.09
Chronic kidney disease	2.80 (2.46–3.18)	<0.01	2.31 (2.02–2.63)	<0.001
COPD	2.23 (1.97–2.53)	<0.01	1.39 (1.22–1.59)	<0.001
Intracranial bleeding	2.80 (2.13–3.68)	<0.01	1.93 (1.46–2.55)	<0.001
Stroke/TIA	2.70 (2.43–3.00)	<0.01	1.74 (1.55–1.95)	<0.001
Malignancy	1.84 (1.63–2.08)	<0.01	1.75 (1.55–1.99)	<0.001
Rheumatoid arthritis	0.97 (0.63–1.50)	0.90	0.92 (0.60–1.42)	0.71
Systemic lupus erythematosus	0.80 (0.30–2.13)	0.66	2.15 (0.79–5.87)	0.13
Sjogren’s syndrome	1.18 (0.69–2.04)	0.55	1.16 (0.66–2.03)	0.60
Ankylosing spondylitis	0.38 (0.09–1.50)	0.166	0.47 (0.12–1.88)	0.29
Psoriasis	1.29 (0.69–2.40)	0.43	1.06 (0.57–1.97)	0.86
PAOD	1.41 (1.13–1.76)	<0.01	0.97 (0.77–1.21)	0.76
Antibiotics use	1.46 (1.33–1.59)	<0.01	1.38 (1.26–1.51)	<0.001

HR, hazard ratio; CI, confidence interval; DVT, deep vein thrombosis; COPD, chronic obstructive pulmonary disease; TIA, transient ischemic attack; SLE, systemic lupus erythematosus; AS, ankylosing spondylitis; PAOD, peripheral arterial occlusion disease; † adjusted for age, sex, hypertension, hyperlipidemia, diabetes, ischemic heart disease, chronic kidney disease, COPD, intracranial bleeding, stroke, malignancy, rheumatoid arthritis, systemic lupus erythematosus, Sjogren’s syndrome, ankylosing spondylitis, psoriasis, PAOD, and antibiotics use.

**Table 4:** DVT Subgroup analysis for the risks of sepsis.

	Non-DVT		DVT			
	N	No. of Sepsis	N	No. of Sepsis	HR (95% CI)	*p*-Value
Age ^a^						
20–39	463	8	521	39	4.24 (1.97–9.16)	<0.01
40–64	2146	150	2330	301	1.97 (1.61–2.41)	<0.001
≥65	3144	688	2902	862	1.68 (1.85–1.52)	<0.01
*p* for interaction <0.01						
Sex ^b^						
Female	3359	470	3327	662	1.78 (1.58–2.00)	<0.01
Male	2394	376	2426	540	1.85 (1.62–2.12)	<0.01
*p* for interaction = 0.68						
Hypertension ^b^						
No	3132	322	3283	564	2.19 (1.91–2.52)	<0.01
Yes	2621	524	2470	638	1.56 (1.38–1.75)	<0.01
*p* for interaction <0.01						
Hyperlipidemia ^c^						
No	4813	718	4876	1011	1.78 (1.62–1.96)	<0.01
Yes	940	128	877	191	1.89 (1.51–2.37)	<0.01
*p* for interaction = 0.54						
Diabetes ^b^						
No	4582	566	4637	869	1.97 (1.77–2.19)	<0.01
Yes	1171	280	1116	333	1.50 (1.28–1.76)	<0.01
*p* for interaction = 0.08						
Ischemic heart disease ^d^						
No	4778	622	4848	948	1.91 (1.72–2.11)	<0.01
Yes	975	224	905	254	1.47 (1.22–1.77)	<0.01
*p* for interaction = 0.06						
Chronic kidney disease ^e^						
No	5377	736	5315	1037	1.82 (1.65–2.00)	<0.01
Yes	376	110	438	165	1.37 (1.05–1.78)	0.02
*p* for interaction = 0.16						
COPD ^f^						
No	5264	708	5320	1059	1.83 (1.66–2.02)	<0.01
Yes	489	138	433	143	1.55 (1.22–1.98)	<0.01
*p* for interaction = 0.38						
Intracranial bleeding ^g^						
No	5679	818	5682	1177	1.82 (1.67–2.00)	<0.01
Yes	74	28	71	25	1.52 (0.80–2.89)	0.20
*p* for interaction = 0.29						
Stroke/TIA ^h^						
No	5071	634	5120	978	1.90 (1.72–2.11)	<0.01
Yes	682	212	633	224	1.47 (1.21–1.78)	<0.01
*p* for interaction = 0.03						
Malignancy^i^						
No	5007	701	5058	1050	1.74 (1.58–1.91)	<0.01
Yes	746	145	695	152	2.15 (1.70–2.72)	<0.01
*p* for interaction = 0.11						
Rheumatoid arthritis ^j^						
No	5691	837	5692	1190	1.79 (1.64–1.96)	<0.01
Yes	62	9	61	12	2.32 (0.82–6.58)	0.11
*p* for interaction = 0.84						
Systemic lupus erythematosus ^k^						
No	5736	NA	5739	NA	1.73 (1.58–1.88)	<0.01
Yes	17	NA	14	NA	4.99 (0.30–83.47)	0.26
*p* for interaction = 0.84						
Sjogren’s syndrome ^l^						
No	5718	842	5713	1193	1.78 (1.63–1.94)	<0.01
Yes	35	4	40	9	2.64 (0.75–9.22)	0.13
*p* for interaction = 0.39						
AS^b^						
No	5739	NA	5739	NA	1.79 (1.64–1.96)	<0.01
Yes	14	NA	14	NA	NA	NA
Psoriasis ^m^						
No	5731	840	5728	1198	1.82 (1.66–1.99)	<0.01
Yes	22	6	25	4	0.69 (0.04–13.49)	0.81
*p* for interaction = 0.03						
PAOD ^n^						
No	5589	813	5570	1154	1.81 (1.65–1.98)	<0.01
Yes	164	33	183	48	1.45 (0.91–2.31)	0.12
*p* for interaction = 0.46						
Antibiotics use ^b^						
No	4056	495	4123	804	1.95 (1.74–2.18)	<0.01
Yes	1697	351	1630	398	1.58 (1.36–1.83)	<0.01
*p* for interaction = 0.03						

^a^ Adjusted for all variables, excluding COPD, systemic lupus erythematosus, Sjogren’s syndrome, ankylosing spondylitis, and psoriasis; ^b^ adjusted for all variables; ^c^ adjusted for all variables, excluding ankylosing spondylitis; ^d^ adjusted for all variables, excluding systemic lupus erythematosus; ^e^ adjusted for all variables, excluding intracranial bleeding, systemic lupus erythematosus, ankylosing spondylitis, and psoriasis; ^f^ adjusted for all variables, excluding systemic lupus erythematosus, and ankylosing spondylitis; ^g^ adjusted for all variables, excluding chronic kidney disease, rheumatoid arthritis, systemic lupus erythematosus, Sjogren’s syndrome, ankylosing spondylitis, and peripheral arterial occlusive disease; ^h^ adjusted for all variables, excluding Sjogren’s syndrome and ankylosing spondylitis; ^i^ adjusted for all variables, excluding Systemic lupus erythematosus and ankylosing spondylitis; ^j^ adjusted for all variables, excluding intracranial bleeding, systemic lupus erythematosus, and ankylosing spondylitis; ^k^ adjusted for age and sex; ^l^ adjusted for all variables, excluding intracranial bleeding, stroke/TIA, systemic lupus erythematosus, ankylosing spondylitis, and psoriasis; ^m^ adjusted for all variables, excluding chronic kidney disease, rheumatoid arthritis, systemic lupus erythematosus, Sjogren’s syndrome, ankylosing spondylitis, and peripheral arterial occlusive disease; ^n^ adjusted for all variables, excluding intracranial bleeding, systemic lupus erythematosus, ankylosing spondylitis, and psoriasis; NA, not available.

## Data Availability

Restrictions apply to the availability of these data. Data were obtained from the National Health Insurance database and are available from the authors with the permission of the National Health Insurance Administration of Taiwan.
